# Severe TBI patients benefit from multi-modal and multi-disciplinary treatments approaches – two exemplary case reports

**DOI:** 10.25122/jml-2019-0016

**Published:** 2019

**Authors:** Kulesza Katarzyna, Tomczak Agata, Michalak Marcin

**Affiliations:** Department of Nerology, St. Wincenty a Paulo Hospital, Gdynia, Poland

**Keywords:** Craniocerebral injury, craniotomy, cerebrolysin, physical rehabilitation, psychological counseling

## Abstract

We aim to demonstrate that multidisciplinary treatment through neurosurgical intervention, pharmacotherapy, rehabilitation, speech, and psychological therapy is the most promising treatment approach for patients with severe craniocerebral injuries. Here we describe two clinical cases who presented with an unexpectedly positive outcome as both patients regained mobility and the ability to function independently after receiving multimodal therapy.

## Introduction

Craniocerebral injuries are a severe challenge for modern medicine. Due to their character, they are characterized by higher mortality than any other trauma [2]. It is estimated that in highly industrialized countries (e.g., the USA and Western Europe) approximately 1.5 million craniocerebral injuries occur annually and mortality is estimated at 15-30 per 100.000 [1]. The heterogeneity of injury types and, consequently, the neurological deficits observed in patients, mandates close cooperation with interdisciplinary medical teams.

### Case 1:

**Figure 1:**
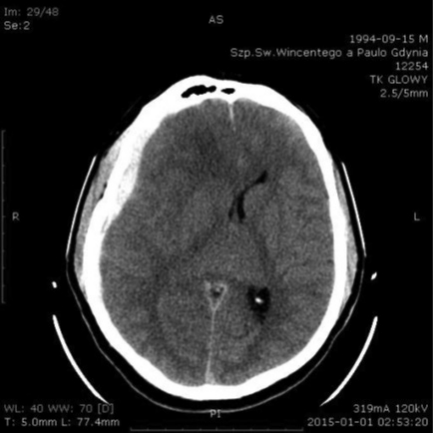
Head CT scan January 1^st^, 2015

A.T. was a 20-year-old male student at the naval academy. He suffered from a head injury during New Year’s Eve 2014. He was admitted to the emergency room, where he presented with quantitative disturbances of consciousness with Glasgow Coma Scale (GCS) sub-scores of 1/4, 1/5 and 2/6 and a total GCS of 4/15. A head CT scan showed an acute, subdural hematoma in the right frontal area with a diameter of 7 millimeters. Brain edema was present as well. The CT scan showed hemorrhagic foci in the left frontal lobe and the pons (Figure 1).

A right-sided craniotomy was performed on January 1st. The stay at the clinic was otherwise uneventful. Nine days later, the patient was transferred to the surgical unit for further management. On admission, the patient was alert, without verbal contact, but he comprehended simple, verbal commands and performed voluntary movements with his right upper limb. Otherwise, the patient was triplegic, with bilateral pyramidal tract signs.

**Figure 2:**
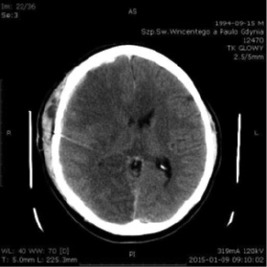
Head CT scan January 9^th^, 2015

A control head CT scan was performed nine days after injury (Figure 2). It revealed a 5-millimeter residual subdural hematoma, focal brain edema, and multiple hypodense areas in the right frontal, parietal and temporal lobes. The patient was consulted by a neurologist; two weeks after the injury, Cerebrolysin was administered with a daily dosage of 30 ml for 37 days. The patient was then transferred to the local neurorehabilitation unit. On admission, the neurological status was stationary. The patient was alert, and he performed only simple tasks. The right upper limb was fully operational, and the other limbs were plegic with bilateral pyramidal tract signs. According to the physiotherapist’s assessment, the patient was bedridden without the ability to maintain an upright position. He required personal assistance in performing activities of daily living (ADL). The psychological examination revealed a behavioral control deficit. The behavioral symptoms were so significant that the Mini-Mental State Exam (MMSE) could not be applied.

During the stay in the rehabilitation department, the patient’s condition gradually improved. On discharge, three months after the injury, he presented no movement deficit. The left upper limb was slightly spastic, and right pyramidal signs were present. However, he was fully ambulatory and completely independent attending to his activities of daily living.

**Figure 3 and 4:**
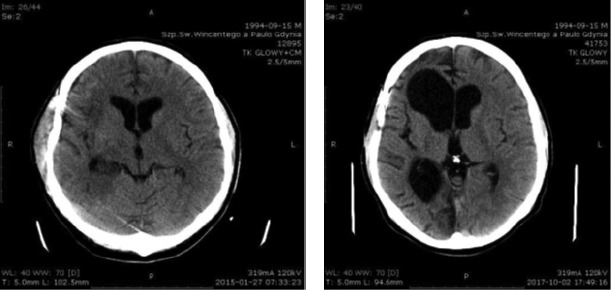
Head CT scans, January 27^th^, 2015 and October 2^nd^, 2017

On psychological assessment, we noticed a significant improvement in cognitive abilities. The patient scored 28 points in the MMSE test which corresponds to his age - average. His behavior had adjusted more to his situation, and there was also a significant improvement in the adequacy of emotional reactions. However, impulsivity and a slight mood deterioration, resulting from the awareness of the limitations associated with the disease persisted. During the following year, the patient was under further outpatient care of the therapeutic team (psychologist, speech therapist, physiotherapist) (Figure 3 and 4).

Further improvement in motor functions has been achieved. However, in order to obtain relative self-sufficiency in instrumental activities of daily living (IADL) the patient required another 12 months of psychological therapy. Currently, the patient is entirely independent with regard to IADL. He was not re-admitted to the Naval Academy but started to work instead. Last year, he was admitted to a drug rehab unit due to marijuana and amphetamine abuse.

### Case 2:

**Figure 5:**
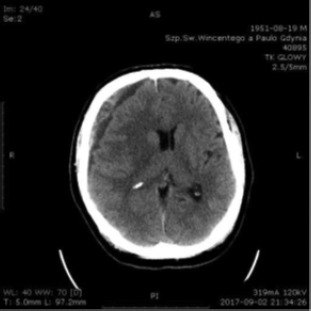
Head CT scan September 2^nd^, 2017

J.W. is a 66-year-old male patient, manager of a large company, with a history of hypertension. He suffered from a head injury during a car accident on July 16th, 2017. Six weeks later he was admitted to the neurosurgery unit and diagnosed with bilateral, subacute, subdural hematomas (Figure 5).

On neurological examination, the patient was conscious, with full verbal contact. He scored 15 points in the GCS but complained of headache scoring 8 out of 10 in the visual analog scale (VAS). Furthermore, he also reported a subjective weakness of the lower limbs.

A bilateral craniotomy was performed. During the four-day stay in the neurosurgery unit, a single epileptic seizure occurred. The patient was transferred to the surgery department, and due to collective epileptic seizures, the patient was consulted by a neurologist and finally transferred to the Neurology Department. On admission, he presented with quantitative disturbances of consciousness, he was drowsy with a psychomotor downturn, oriented to time and place. Dysarthria and central lesions to the left facial nerve were present. The patient was quadriparetic (MRC 3/5 – Medical Research Council Scale of Muscle Strength) with decreased muscle tone in all limbs.

**Figure 6:**
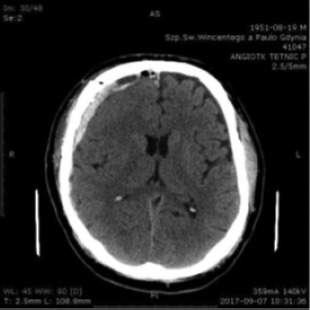
Head CT scan, September 7, 2017

A control head CT scan on September 7, 2017, showed bilateral hematomas in the frontotemporal areas (Figure 6).

The patient was treated with Valproic acid to control the seizures and, in addition, Cerebrolysin was administered for a total of 29 days, with a daily dosage of 30ml. Furthermore, the patient has received rehabilitation therapy and psychological counseling during the stay in the neurology unit.

According to the psychological assessment, fluctuating qualitative and quantitative disturbances of consciousness, mainly escalating in the evening, were present. During the second week of hospitalization, the patient was auto- and allo - psychically disoriented. Occasionally, he was delusional. At night, constant iv infusions of benzodiazepines were sometimes required. During the day, the patient manifested serious attention disturbances. His speech was slurred. Periodically, he presented disinhibition, variability in affect motivation.

On discharge, he needed to continue psychological therapy. He was able to walk with crutches, and he required some assistance with ADL.

About two months after craniotomy, the patient was re-hospitalized.

**Figure 7:**
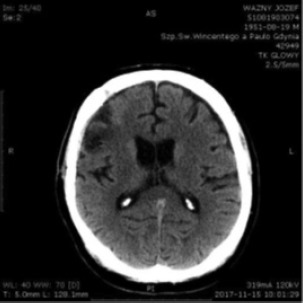
Head CT scan, 15^th^ November, 2017

A control head CT scan taken November 15th revealed regression of the brain hematomas and cerebral edema (Figure 7). On neurological examination, he presented left-side pyramidal signs and psychomotor downturn. The patient required some assistance with IADL. No certain epiform waves were recorded in the EEG. He is treated with sodium valproate at 2000 mg daily dose. No epileptic seizures have been observed lately.

The patient, nine months after the injury, continues neuropsychological therapy, remains independent in the ADL and plans on returning to work.

## Discussion

The forces which are acting during a head injury, trigger a set of factors causing damage to the brain structure as well as acute disturbances of brain function and eventually further processes leading to secondary brain damage [3]. Furthermore, craniocerebral trauma causes mechanical damage to the brain tissue and blood vessels as well as to the nervous system leading to dysfunction of vasoreactivity. In addition, epidural or subdural hematomas, fractures of the skullcap or the skull base may take place, sometimes with accompanying rupture of the dura mater and consequently leakage of cerebrospinal fluid [4,5]. Craniocerebral injuries are among the most common reasons for long-term exclusion from socio-professional life. Restoring the patient to pre-injury levels of functioning requires the implementation of various measures: surgical treatment, pharmacotherapy, psycho -, logo - and physiotherapy. Nevertheless, it should also be noticed that sociodemographic factors such as age, sex, economic status, place of residence, and professional activity, have a significant impact on the clinical outcome, especially in the post-hospitalization phase.

Cerebrolysin is a neurotrophic drug with a modulatory effect on brain plasticity. It also stimulates endogenous production of neurotrophic factors (NTFs) in brain tissue and triggers synaptic remodeling as well as modulation of synaptic transmission post-injury.

## Conclusion

Cerebrolysin is a rational choice for pharmacotherapy in severe TBI patients which is also documented by the two cases presented above.

What makes these two cases unique is the fact that we decided for a long-term therapy - 37 days and 29 days, respectively - because we observed continuous and dynamic improvement throughout treatment and we associate the functional recovery with the administration of Cerebrolysin.

In contrast, patients treated in our institution for similar forms of TBI who do not receive Cerebrolysin generally recover more slowly and most of them cannot regain a satisfactory status of independence.
